# High Strength Die-Attach Joint Formation by Pressureless Sintering of Organic Amine Modified Ag Nanoparticle Paste

**DOI:** 10.3390/nano12193351

**Published:** 2022-09-26

**Authors:** Xingwang Shen, Junjie Li, Shuang Xi

**Affiliations:** 1 College of Mechanical and Electronic Engineering, Nanjing Forestry University, Nanjing 210037, China; 2 Shenzhen Institute of Advanced Electronic Materials, Shenzhen Institute of Advanced Technology, Chinese Academy of Sciences, Shenzhen 518100, China

**Keywords:** Ag nanoparticle paste, pressureless sintering, surface modification, high-strength joint, die-attach

## Abstract

Sintered silver (Ag) die-attach has attracted much attention in power systems with high power density and high operating temperature. In this paper, we proposed a novel surface modification method for Ag nanoparticles with organic amines as a coating agent for enhancing the pressureless sintering performance. This work systematically introduced the Ag nanoparticle modification process, Ag paste preparation, and sintering process and compared the changes in the sintering performance of Ag nanoparticles after modification with four different alkyl chain lengths of amines. The study showed that the sintered films of Ag nanoparticle pastes modified with *n*-octylamine (NOA) can achieve the lowest resistivity of the sintered film and the highest shear strength of the bonded joints. The resistivity of the sintered Ag film is affected by the grain size and microscopic morphology, and the strength of the bonded joints is also related to the sintering density and the amount of organic residues. The thermal behavior of the Ag particles coated with different amines is measured by thermal analysis. Finally, the mechanism of NOA-modified Ag nanoparticles to improve the sintering performance is proposed. This study can provide effective data and theoretical support for the further promotion and application of nano-Ag pressureless sintering.

## 1. Introduction

With the rapid development of electric vehicles, smart grids, wireless communications, and other fields, silicon-based semiconductor devices have been unable to meet the high temperature, high power, and other service requirements in terms of its operation junction temperature which is below 200 °C and voltage blocking capabilities. Wide band gap (WBG) semiconductors, such as SiC and GaN, with the advantages of high switching frequency, high breakdown field strength, high bonding energy, high thermal conductivity, high temperature operation, and radiation resistance, have become the developing direction of new generation power devices [1,2,3,4,5]. At the same time, its packaging methods and interconnect materials become a real challenge in nowadays power packaging technology, for the materials used in high temperature electronic packaging must be able to withstand such conditions. Traditional tin-based solders are not suitable for the development needs of wide-band-gap semiconductor because the interconnect performance declines dramatically at service temperatures above 150 °C [6,7,8,9,10,11,12]. Diverse high temperature die attach materials have been studied. Gold-based and zinc-based solder alloys have limited their widespread application in power device die attachment due to their high cost, brittleness of intermetallic compounds (IMCs), and poor corrosion resistance. Although transient liquid phase bonding (TLP) can achieve bonding at lower temperatures (250–300 °C) and shorter times and obtain higher remelting temperatures, the reliability of its bonding is also vulnerable to intermetallic compound (IMCs) brittleness [13,14,15,16,17,18,19].

At present, Ag sintering is a promising technology for power device interconnects. Ag paste has low temperature sintering capability and its sintered structure has high temperature resistance as well as excellent electrical conductivity, thermal conductivity, and high reliability, which can meet the requirements of reliable electrical connection and heat dissipation of high power and high junction temperature chips [20,21,22,23,24]. According to previous reports [25,26], different sintering temperatures, pressures, heating times, and sizes and shapes of the Ag particles all affect the sintered Ag microstructure and sintering performance. The classical sintering process can be carried out at temperatures ranging from 200 to 300 °C with the sintering pressure range from 10 MPa to 40 MPa [27]. However, the pastes in which the powder particles are a few to several micrometers in size are processed at temperatures above 250 °C and at pressures higher than 10 MPa, which may increase the risk of chip damage and increase the process difficulty of chip fabrication. Therefore, the development demand for pressureless sintering Ag technology has become an increasing urgent need. According to the size effect, the surface energy of Ag particles will increase dramatically when they reach the nanometer scale, and the diffusivity of Ag atoms will be significantly enhanced during the sintering process. Therefore, nano-Ag pastes have become the primary material for the development of pressureless die-attach technology. Meanwhile, with the development of nanotechnology, the synthesis process of Ag nanoparticles has gradually matured, and the manufacturing cost has been well controlled, which provides the necessary technical support for the industrial application of nano-Ag paste. However, smaller Ag nanoparticles can easily form micron-sized agglomerates at room temperature due to their overactive surface energy, which reduces the sintering drive of the Ag paste during the sintering process and leads to degradation of the sintering performance. Therefore, how to avoid the spontaneous agglomeration of Ag nanoparticles at room temperature deserves further study. Based on the present state of research [28], the main method to prevent nano-Ag agglomeration is to add a coating agent during the synthesis of Ag monomers with Ag precursors. The types of coating agents are generally classified as polymers and small molecule compounds. Generally, polymers with carboxylate, amino, or hydroxyl functional groups possess high decomposition temperature, such as polyacrylic acid (PAA) [29,30] and polyvinylpyrrolidone (PVP) [31,32,33], and organic matter will remain after sintering and bonding, which influence their sintering quality. Small molecule compounds are always with long alkyl chains and polar heads, such as alkanethiols [34,35,36], alkylamines [37], and carboxylic acids [38], which present a relatively wide range of decomposition temperature. Therefore, choosing the small molecule compound coating agent with a suitable decomposition temperature becomes the critical issue to enhance the anti-agglomeration property and the sintering performance. At the same time, in general, the synthesis of Ag monomers also suffers from the long time required and the instability of the product.

At present, there are few studies on the surface modification of commercial Ag nanoparticles. Based on the demand, this work proposes a novel method for surface modification of commercial Ag nanopowders by organic amines, which was then prepared into nano-Ag paste for pressureless die-attach application. Four kinds of amines (*n*-octylamine, dodecylamine, hexadecylamine, and octadecylamine) were utilized to modify nano-Ag powders by washing, dispersing, and freeze-drying processes, and sintering and bonding experiments were conducted to character the microstructure, shear strength, resistivity, and cross-sectional porosity of these Ag pastes. Based on the experimental results, we found that the performance of the amine-modified Ag nanoparticles with different chain lengths differed dramatically after sintering. This is related to the difference in their boiling points. Then, the mechanism of organic amine modified Ag nanoparticles to enhance low temperature sintering properties was proposed.

## 2. Materials and Methods

### 2.1. Materials

The commercial nano-Ag powder used in this paper was purchased from Guangdong Lingguang New Material Co., Ltd. (Zhaoqing, Guangdong, China), Octylamine, dodecylamine, hexadecylamine, octadecylamine, ethanol, *tert*-butanol, and polyol ether organic solvents in Ag paste were purchased from Shanghai Aladdin Bio-Chem Technology Co., Ltd. (Shanghai, China), and all reagents were of analytical grade without further purification. The upper and lower substrates are DBC (Direct Bonded Copper) ceramic substrates with Ni/Au surface coating layer, purchased from Jiangsu Ferrotec Semiconductor Co., Ltd. (Yancheng, Jiangsu, China).

### 2.2. Surface Modification of Ag Nanoparticles

In this paper, four amines with different alkyl chain lengths are introduced as coating agents for Ag nanoparticles, which are *n*-octylamine (C_8_H_19_N, NOA, boiling point ~179 °C), dodecylamine (C_12_H_27_N, DDA, boiling point ~258 °C), hexadecylamine (C_16_H_35_N, HDA, boiling point ~322 °C), and octadecylamine (C_18_H_39_N, ODA, boiling point ~349 °C). The coating agents were used to prevent agglomeration of nanoparticles during preservation and before sintering. The detailed process of surface modification is described as follows. Firstly, 2 g of commercial Ag nanoparticles were mixed with 30 mL of ethanol and then subjected to ultrasonic dispersion and freezing centrifugation to remove organic impurities from the surface of the Ag particles. The cleaning process was conducted 3 times for better removal of surface impurities. Then, 30 mL of ethanol, and 0.04 g of organic amines were mixed with the cleaned Ag powder, and the excess organic amines were removed by ultrasonic dispersion and freezing centrifugation. Next, the nanoparticles were dispersed into a mixture of *tert*-butanol and ethanol, placed in a refrigerator for 6 h, and then freeze-dried for 10 h. Finally, the obtained surface-modified Ag nanoparticle powder was collected for further use.

### 2.3. Modified Nano-Ag Paste Preparation and Bonding Process

**Figure 1:**
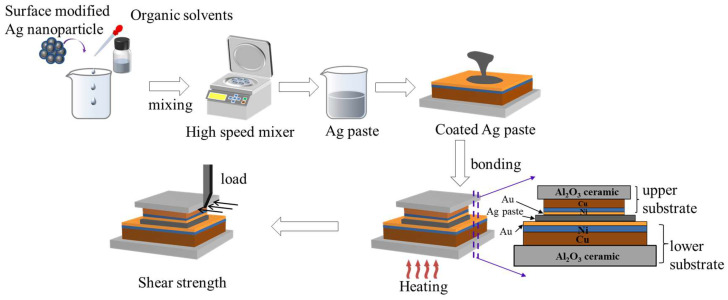
Nano-Ag paste preparation and pressureless sintering bonding process.

The four amine-modified Ag nanopowders were mixed with organic solvents as respective, and the homogeneous nano-Ag paste was formed by high-speed stirring. The paste had an Ag content of 85 wt.% to achieve a suitable viscosity for the dispensing process. The organic solvents in the Ag paste are ethylene glycol, terpineol and polyethylene glycol 200, which can be completely evaporated at 250 °C. The pastes prepared from unmodified Ag nanoparticles and Ag nanoparticles modified with different amines were defined as Ag-0, Ag-NOA, Ag-DDA, Ag-HDA, and Ag-ODA, respectively. The bonding substrate is DBC ceramic substrate with 5-μm thick Ni layer and 70-nm thick Au layer coated on the surface of the Cu layer of the DBC. The upper and lower substrate sizes are 3 × 3 × 0.3 mm and 6 × 6 × 0.3 mm to simulate practical application scenarios. The DBC substrates were removed from the surface contaminants on the substrate by sonication in ethanol for 5 min before use. The Ag paste was coated on the surface of the lower DBC substrate by a dispensing process, and then the upper substrate was placed on the Ag paste to form a sandwich bonding structure. Figure 1 shows a schematic diagram of the Ag paste preparation and bonding process. The interconnected joints in this experiment were achieved by pressureless sintering of the prepared sandwich samples under environmental conditions. During the bonding process, the sample was placed on a heating plate and heated from 30 °C to 250 °C at a steady heating rate of 16 °C/min, held for different times (10 min, 30 min, 60 min) and then cooled naturally. The heating curve of the sintering process is shown in Figure S1.

### 2.4. Measurement and Characterization

To examine the coating effect of Ag nanoparticles, Fourier transform infrared absorption spectroscopy (FT-IR, Invenio R, Beuker, Karlsruhe, Germany), energy dispersive X-ray energy spectroscopy (EDS, FEI Nova Nana SEM 450, FEI, Hillsboro, OR, USA), and X-ray photoelectron spectroscopy (XPS, Thermo Scientific Escalab 250xi, Thermo Fisher Scientific, Waltham, MA, USA) were used for surface composition analysis. The resistivity of the sintered Ag films was measured by a 4-point probe system (Loresta-GP MCP-T600, Mitsubishi Chemical, Kanagawa, Japan). Physical phase identification of sintered Ag films was conducted by X-ray diffractometry (XRD, D8 ADVANCE A25, Beuker, Karlsruhe, Germany). The bond strength of Ag-Au joints was evaluated by a chip shear tester (DAGE4000, Nordson DAGE, Aylesbury, UK), with a shear head speed of 100 μm/s and a shear height of 50 μm from the lower substrate surface. The average value of shear strength was calculated after testing 6 joints in each group. Clear and accurate cross-sectional structures of the bonded joints were prepared by a grinding and polishing machine (Tegramin, Struers, Ballerup, Denmark). The microscopic morphology of commercial Ag nanoparticles was observed by transmission electron microscopy (TEM, JEM-ARM200F, JOEL, Tokyo, Japan). The sizes of commercial Ag nanoparticles were measured by image processing software (Nano Measurer version 1.2, Shanghai, China). The structural features of Ag nanoparticles, sintered Ag films, and bonded joints were characterized by field emission scanning electron microscopy (FE-SEM, FEI Nova Nana SEM 450, FEI, Hillsboro, OR, USA). The porosity of the joint cross-section was calculated with the Image pro Plus (version 6.0, Media Cybernetics, Silver Spring, MD, USA) software. The thermal behavior of different amine-modified Ag nanoparticles was measured using a thermogravimetric analyzer (TGA/DSC 2, Mettler Toledo, Greifensee, Switzerland) at a holding time of 60 min at 250 °C. The measurements were conducted at an air flow rate of 100 mL/min and a heating rate of 10 °C/min.

## 3. Results and Discussion

### 3.1. Characterization of Commercial Ag Nanoparticles

**Figure 2:**
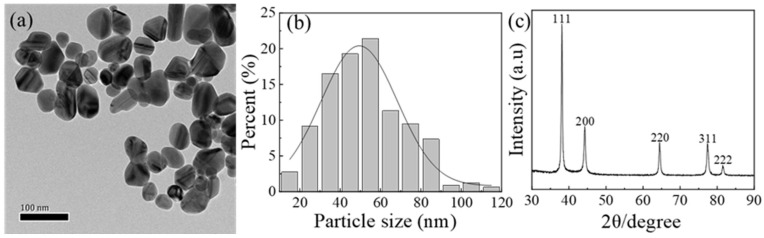
Morphology, size and physical phase analysis of commercial Ag nanoparticles (**a**) TEM image (**b**) Size distribution (**c**) XRD diffraction image.

Due to the problems of unstable product batches, unknown surface organics. and poor sintering performance of commercial Ag nanoparticles, we introduced a nano-Ag surface modification process to improve the stability and sintering performance of Ag nanoparticle pastes. Figure 2a shows the transmission electron micrograph (TEM) of commercial Ag nanoparticles with spherical and quasi-spherical shapes. Figure 2b shows the particle size distribution of Ag nanoparticles. The average particle size is approximately 50 nm, and the distribution is concentrated between 30 nm and 80 nm. Figure 2c shows the XRD diffraction pattern of commercial Ag nanoparticles, indicating a high purity with no other impurities.

### 3.2. Analysis of the Effect of Organic Amine Modification on the Surface of Ag Nanoparticles

**Figure 3:**
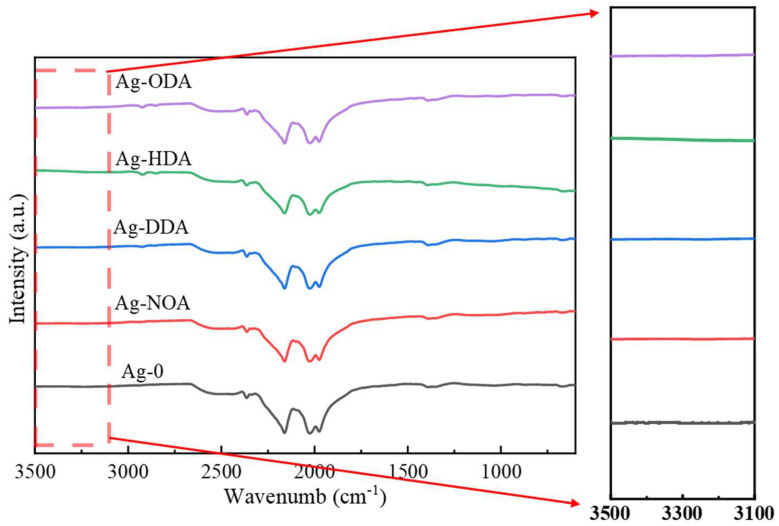
FT−IR spectra of different organic amine modified Ag nanoparticles.

**Figure 4:**
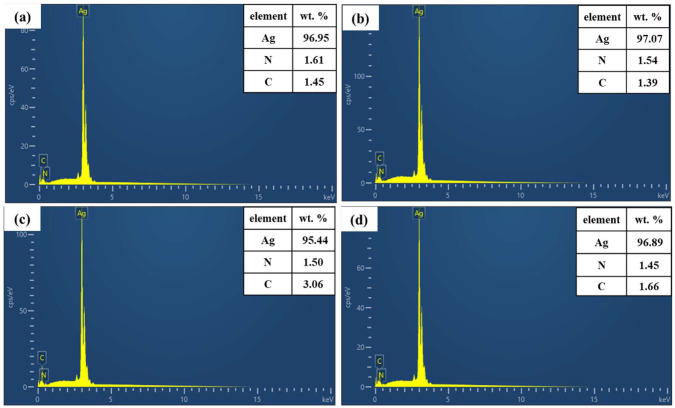
EDS energy spectra of Ag nanoparticles modified with different organic amines (**a**) NOA (**b**) DDA (**c**) HDA (**d**) ODA.

**Figure 5:**
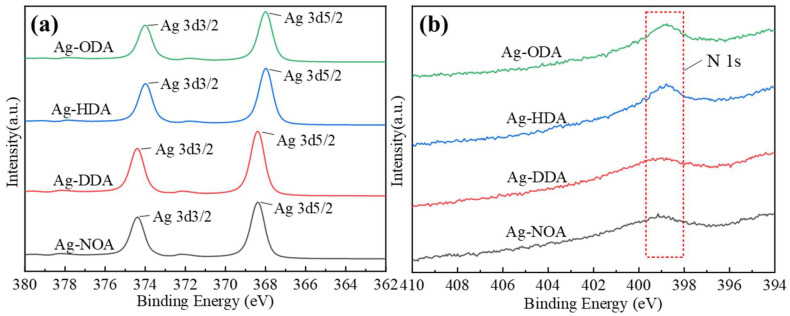
XPS high-resolution spectrum of Ag nanoparticles modified with different organic amines (**a**) Ag 3d spectrum (**b**) N 1s spectrum.

Figure 3 shows the FT−IR spectra of different organic amine modified Ag nanoparticles. In fact, *n*-octylamine, dodecylamine, hexadecylamine, and octadecylamine are all primary amines and will have two moderate intensity stretching vibration peaks at 3500–3100 cm^−1^, while no such stretching vibration peaks can be found on the amine-modified Ag powder. Combined with other related reports [35,39,40], it can be inferred that –NH_2_ undergoes coordination reactions with Ag atoms to form Ag–N bonds, so that the characteristic peaks of N–H cannot be detected. Meanwhile, in the FT−IR spectra of NOA, DDA, HDA, and ODA treated Ag powders, we find a faint stretching vibration peak near 2750 cm^−1^, which is a stretching belonging to the –CH_2_– group on the amino chain, which further proves the presence of amines. Figure 4a–d show the EDS energy spectra for NOA, DDA, HDA, and ODA modified Ag powders, respectively. The characterization results clearly show the characteristic peaks of elemental N, providing evidence that the organic amines were not removed during the cleaning process. However, we found that the proportion of N elements varied in different amine-modified Ag powders, which may be related to the number of carbon chains of the amine. Specifically, longer carbon chains will have a more significant spatial site resistance effect, which could weaken the ability to coordinate with Ag atoms [41]. In addition, in order to further observe the elemental and chemical states on the surface of organic amine-modified Ag nanoparticles, different modified Ag particles are analyzed by XPS. The binding energies in the XPS spectrum of the modified Ag particles shown in Figure 5 are calibrated by using the binding energy of C 1s (284.8 eV). Figure 5a shows the high resolution spectrum of Ag 3d, the splitting of the 3d double peak spanning 6 eV, which shows that the Ag nanoparticles are metallic in nature [42]. Meanwhile, no peaks related to Ag oxide compounds (367.3 eV for AgO and 367.7 eV for Ag_2_O) are found [43]. Interestingly, the Ag 3d5/2 spectrum binding energy of Ag particles modified with NOA and DDA shifted to a higher binding energy (368.4 eV), while the Ag 3d5/2 spectrum binding energy of Ag particles modified with HDA and ODA decreased (368.0 eV), compared to the Ag 3d5/2 standard number binding energy (368.2 eV). The main reason for this occurrence is the transfer of electrons due to the interaction of organic amine and Ag nanoparticles, which is similar to other related reports [42,44,45,46]. Figure 5b shows the N 1s high-resolution spectrum, which provides conclusive evidence for the organic amine coating on the surface of the Ag nanoparticles, and the weaker peak intensity may be caused by the low organic amine content. In order to further confirm the absence of Ag oxide compounds in the organic amine modified Ag powder, we conducted the analysis by XRD, as shown in Figure S2. The above results show that all four different organic amines can form effective coating layers on the surface of Ag nanoparticles.

### 3.3. Resistivity of Ag Paste Sintered Films

**Figure 6:**
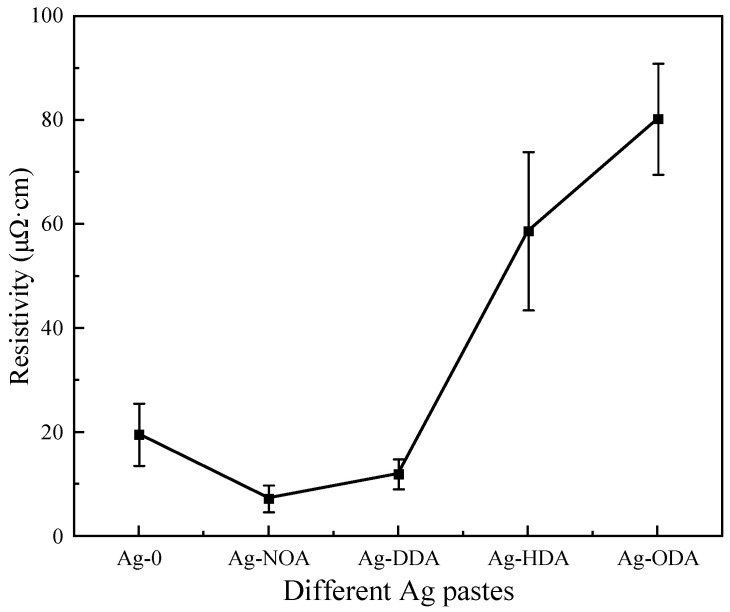
Resistivity of 30 min films sintered with different organic amine modified Ag pastes.

Electrical resistivity is a critical parameter for die-attach materials and related to the current handling capability and electrical power loss [47]. Different amine modified nano-Ag pastes were sintered at 250 °C for 30 min to prepare the sintered films, and the effect of the amine type on the resistivity was studied, as shown in Figure 6. The film prepared with Ag-NOA has the lowest resistivity of only 7.31 μΩ·cm, which is approximately four times higher than that of bulk silver (1.6 μΩ·cm). The resistivity of the film prepared with Ag-DDA increased to 12.02 μΩ·cm, but it was still lower than that of the films prepared with Ag-0 (19.63 μΩ·cm). However, the resistivity of the films prepared by Ag-HDA and Ag-ODA is significantly increased to 58.66 μΩ·cm and 80.16 μΩ·cm, respectively, which are much higher than those of the films prepared by Ag-0. The results show that the type of amine has a significant effect on the film resistivity. NOA modification has the highest conductivity, while HDA and ODA modifications could weaken the conductivity.

**Figure 7:**
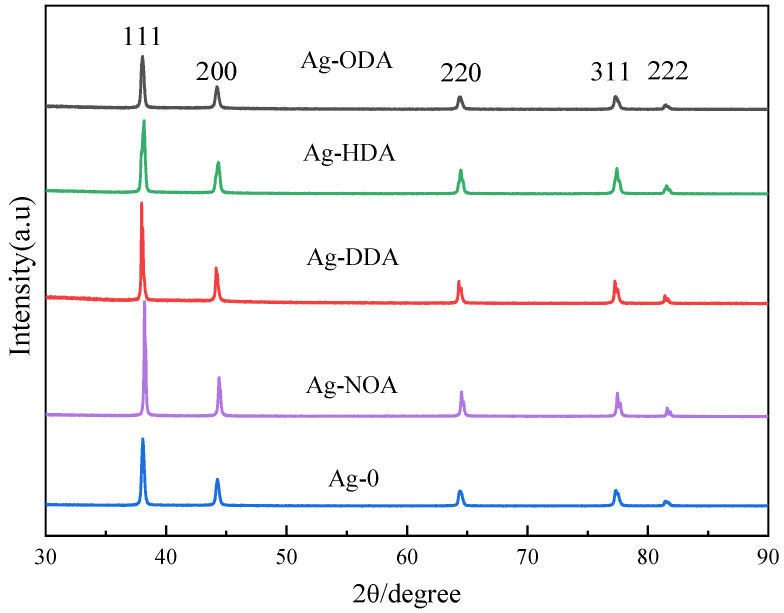
XRD diffractograms of 30 min films sintered with different organic amine modified Ag pastes.

**Figure 8:**
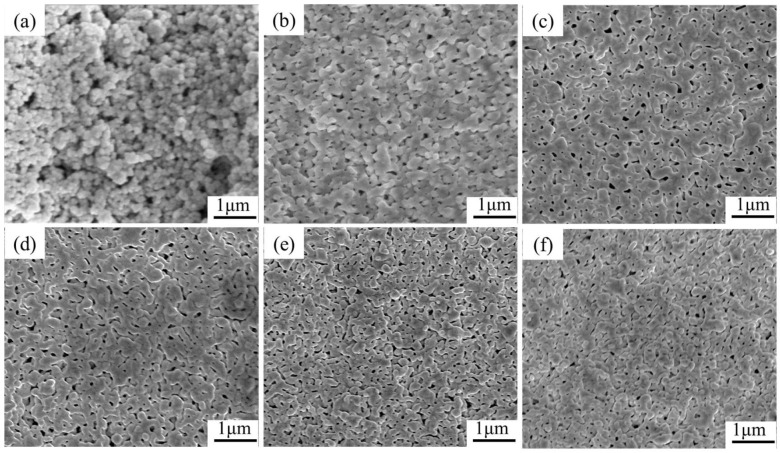
SEM images of commercial Ag nanoparticles at room temperature and different organic amine modified Ag pastes sintered for 30 min (**a**) commercial Ag nanoparticles (**b**) Ag-0 (**c**) Ag-NOA (**d**) Ag-DDA (**e**) Ag-HDA (**f**) Ag-ODA.

To investigate the influence mechanism on resistivity, the crystal structures and surface morphologies of the sintered films of different amine modified Ag pastes were characterized by XRD and SEM, as shown in Figure 7 and Figure 8. There are five main diffraction peaks marked in Figure 7, namely (111), (200), (220), (311), and (222), which correspond to the crystalline plane of the pure Ag phase. These XRD diffraction peaks are consistent with the standard powder diffraction card (JCPDS No. 03-065-2871) for the Ag face-centered cubic crystal structure, and no other diffraction peaks of impurities are detected. The sintered Ag films prepared with Ag-NOA and Ag-DDA have sharp peaks, which indicates a high degree of crystallization of the film structure and the formation of well-crystallized Ag films. However, the sintered Ag films prepared with Ag-HDA and Ag-ODA show relatively weak peaks, indicating a low degree of crystallization of the film structure. In addition, we calculated the grain size of the particles in each Ag film using the Debye–Scherrer formula, as shown in Table 1. It can be clearly found that the grain size of Ag-NOA sintering (55.0 nm) is much larger and more crystalline than that of Ag-0 (35.66 nm) at a 2θ value of approximately 38°. The grain size of Ag-DDA (44.29 nm) is slightly larger than that of Ag-0. However, the grain sizes of both Ag-HDA and Ag-ODA are smaller than that of Ag-0, which further confirms the above analysis for crystallinity.

Figure 8a shows the SEM images of commercial Ag nanoparticles at room temperature, showing partial spontaneous agglomeration, which is a common phenomenon of metal nanoparticles due to the size effect. Figure 8b–f show SEM images of the films prepared from Ag-0, Ag-NOA, Ag-DDA, Ag-HDA, and Ag-ODA, respectively. It can be observed that the microstructure of the films has significant differences in sintering morphology, particle size, and degree of contact. The Ag-0 prepared films show an irregular crystal structure, uneven size of sintered particles, poor state of particle connection, and less sintered neck, indicating insufficient sinter formation. The film prepared by Ag-NOA formed a smooth surface and a uniform and continuous microporous structure, which means that it is already in a relatively adequate sintered state, consistent with the lower resistivity of the Ag-NOA film. Although the Ag film prepared by Ag-DDA also has a relatively dense sintering structure, the Ag particles are still not well connected in some areas, which may account for the increased resistivity. For the Ag films prepared by Ag-HDA and Ag-ODA, we can clearly observe that the degree of connection between Ag nanoparticles is significantly reduced and there are still some Ag particles in an isolated state, which indicates that sufficient sintering has not occurred, resulting in a rapid increase in resistivity.

**Figure 9:**
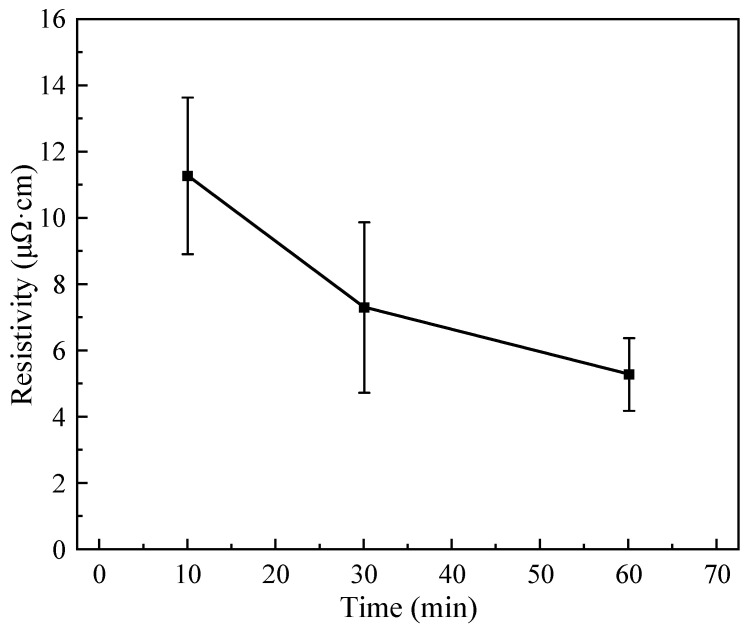
Resistivity of Ag-NOA films for different holding times.

Combining the obtained results, it can be revealed that the Ag-NOA film has the lowest resistivity and the best sintering density. Then, the film was prepared by sintering Ag-NOA at 250 °C for 10, 30, and 60 min to investigate the effect of holding time on resistivity, as shown in Figure 9. The film resistivity after 10 min of holding is 11.28 μΩ·cm, which is approximately seven times higher than that of bulk Ag. When the holding time is increased to 30 min, the resistivity decreased to 7.31 μΩ·cm. When the holding time reaches 60 min, the film resistivity continues to decrease to 5.3 μΩ·cm, which is only about three times the resistivity of bulk Ag and can meet the needs of industrial applications. The above experimental results show that the resistivity decreases with the increase of holding time, while the decrease trend slows down after 30 min sintering.

**Figure 10:**
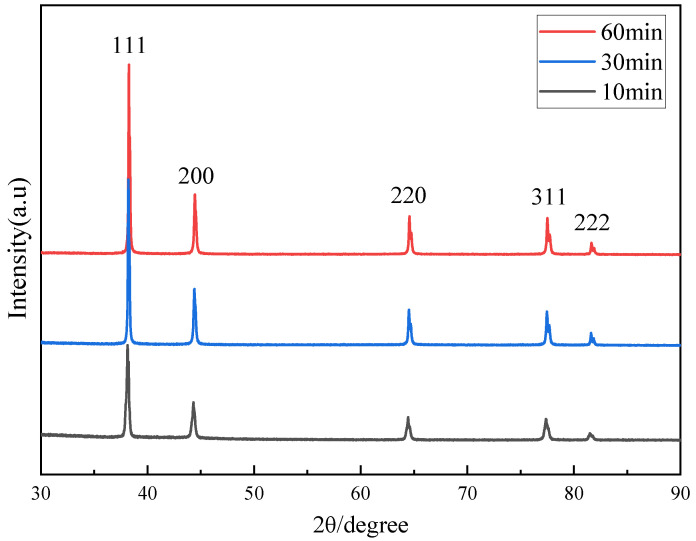
XRD diffraction patterns of Ag-NOA films at different holding times.

**Figure 11:**
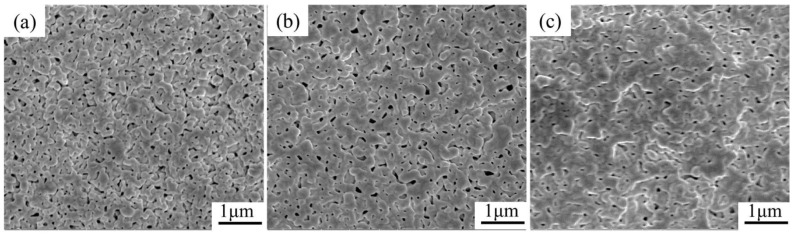
SEM images of Ag-NOA films with different holding times at 250 °C (**a**) 10 min (**b**) 30 min (**c**) 60 min.

In order to explore the mechanism of holding duration on resistivity of Ag-NOA film, the crystal structure and surface morphology of Ag film were characterized by XRD and SEM, as shown in Figure 10 and Figure 11. The five main diffraction peaks marked in Figure 10, similar to the XRD characterization above, correspond to the crystalline plane of the pure Ag phase, and no impurity diffraction peaks were detected. With a continuous increase in the holding time, the diffraction peaks of the Ag films become sharper, which indicates an increasing degree of crystallization of the film structure. We calculated the particle size of Ag nanocrystals in Ag-NOA film at different holding times with the Debye–Scherrer formula, as shown in Table 2. With the increase of sintering time, we find that the particle size increases, which is consistent with the XRD diffraction peak analysis.

The surface morphology of Ag-NOA sintered film with different holding times was characterized by SEM, as shown in Figure 11. With the increase of holding time, the sintering of Ag nanoparticles gradually becomes adequate. The microstructure is looser after holding for 10 min, and the Ag particles are irregular in shape and size, with a low degree of connection. As the holding time increases to 30 min, the sintered connection of Ag particles increases, and a larger sintered neck is formed. After a hold time of 60 min, the independent Ag particles have disappeared and the Ag particles are in good contact with each other, forming a denser sintered network structure. At the same time, the surface pores decreased, indicating that the Ag particles have achieved sufficient sintering. Therefore, increasing the holding time can promote the adequate sintering and diffusion of Ag particles to form a uniform and dense sintering structure, resulting in a lower resistivity of the film.

It can be concluded from the above analysis that highly conductive Ag films can be obtained by selecting suitable amine modified Ag nanoparticles for the preparation of Ag paste. The microstructure of the film prepared by Ag-NOA is uniform and dense with a large Ag crystal composition when held at 250 °C for more than 30 min, but some pores still exist, which cannot be avoided during the sintering process [48,49]. It should be noted here that the particle size of Ag nanoparticles calculated with XRD data belongs to primary particles. However, the particle size shown in SEM is formed by aggregated particles (secondary particles) [41].

### 3.4. Shear Strength of Bonded Joints

**Figure 12:**
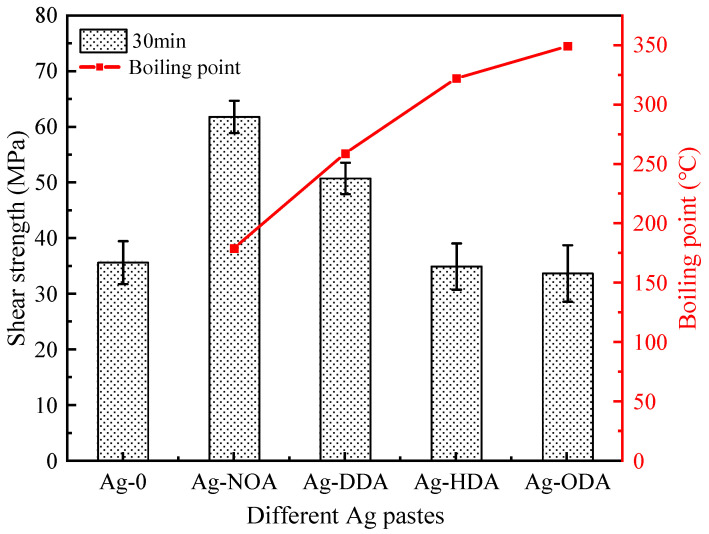
Shear strength of bonded joints with different organic amine modified Ag pastes sintered for 30 min.

In order to evaluate the effect of different amine-modified Ag nanoparticles on the bond strength, a shear strength test was conducted on these samples. Figure 12 shows the shear strength of the joints after 30 min of sintered bonding with different amine modified Ag pastes, indicating that the use of the appropriate chain length amine is essential for the formation of high-strength joints. The average shear strength of Ag-0 bonded joints without the introduction of organic amines is 35.7 MPa. The average shear strength of the bonded joints with Ag-NOA is substantially increased to 61.8 MPa, indicating that the NOA-modified Ag particles can significantly enhance the bond strength of the joints. However, for the base Ag-DDA paste, the average shear strength of the bonded joints decreases to 50.78 MPa, which may be related to the increase in the number of carbon chains. The average shear strengths of Ag-HDA and Ag-ODA bonded joints are 34.98 MPa and 33.73 MPa, respectively, and this result was similar to the strength of unmodified joints. In fact, under the same conditions, the higher the number of carbon chains of the same kind of organic substance, the higher its boiling point. The boiling points of the four amines used in this paper increase with the number of carbon chains. Combined with the above analysis, we speculate that the boiling point of the amine may influence the bonding strength of the modified Ag paste, indicating that the modification of low boiling point amine can enhance the bonding performance. The thermal analysis of the modified Ag particles is further discussed in the following mechanistic explanation.

**Figure 13:**
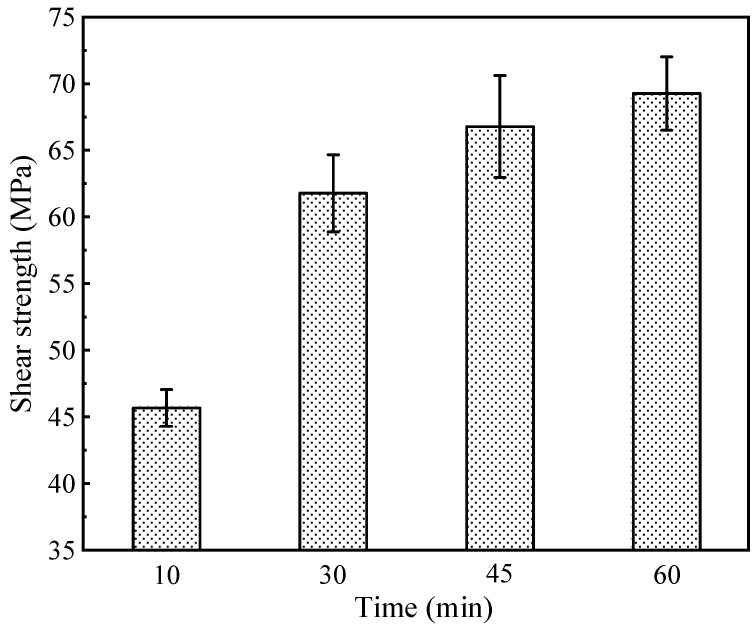
Strength of Ag-NOA bonded joints at different holding times.

The shear strength test above shows the optimal bonding performance of Ag-NOA joints. To investigate the effect of Ag-NOA on the bonding strength at different holding times, we added comparative experiments with holding times of 10 min, 45 min, and 60 min, as shown in Figure 13. The joint strength increases sharply when the holding time increases from 10 min to 30 min, and then increases slightly after 30 min and finally reaches the maximum value of 69.3 MPa at 60 min. The above results indicate that joint strength is positively correlated with holding time. The increase in sintering time leads to an increase in joint strength, which is consistent with existing reports [25,50,51]. Meanwhile, in order to investigate the difference of holding time on the shear strength of the four amine-modified Ag paste bonded joints, a comparison experiment was conducted, and the joint strength results are shown in Figure S3.

**Figure 14:**
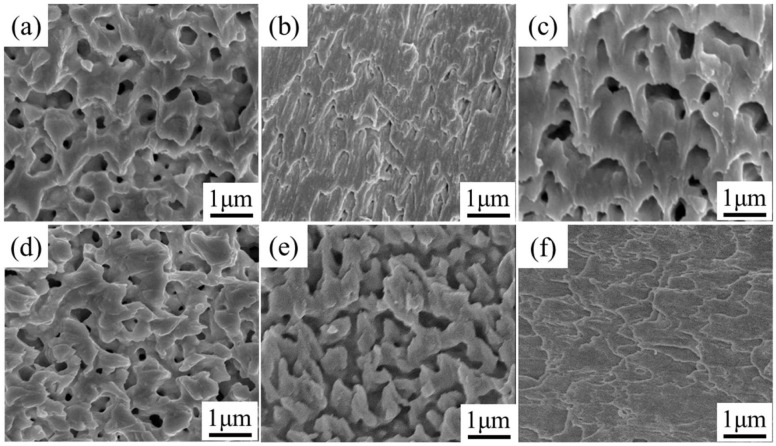
Fracture surface morphology of different modified Ag paste bonded joints (**a**) Ag-0 (**b**) Ag-NOA (**c**) Ag-DDA (**d**) Ag-HDA (**e**) Ag-ODA (**f**) Ag-NOA with 60 min holding time.

The fracture surface morphology of the joint is further characterized to investigate the shear strength with different modification agents. Figure 14a–e show the SEM images of the fracture surfaces of bonded joints sintered with different Ag pastes for 30 min. The Ag-0 joints form fewer triangular ductile stretches of different orientations between the Ag particles. The Ag layers at both the Ag-NOA and Ag-DDA sections tilt at a large angle along one direction, forming a plastic fracture. The Ag-NOA shows greater plastic tensile deformation and a denser sintered structure, indicating a very strong bond between the sintered Ag structure and the DBC substrate. The Ag-DDA exhibits a denser porous structure and a large honeycomb-like structure with tensile deformation. However, the plastic deformation of the Ag-HDA fracture surface is significantly reduced and is no longer sharp, showing a similar morphology to that of the Ag-0 joint. This indicates that the sintering ability of the modified Ag paste is weakened after modification with HDA. The nanoparticles in the fracture surface of the Ag-ODA joint do not undergo large-scale deformation and the fracture position is close to the interface of the DBC substrate. Compared to other joints, the conversion from cohesive damage to adhesive damage is observed. This phenomenon may be related to the ODA organic residues in the bonded structure, which prevents the sintering diffusion between the Ag particles and results in weakening of bonding strength. Therefore, the results of the fracture surface analysis are consistent with the measuring results of shear strength.

To explore the reasons for the difference in strength of Ag-NOA paste with different holding times, SEM images of the fracture surface of Ag-NOA joints with holding times of 60 min are observed, as shown in Figure 14f. It is found that the sections at 60 min have a tighter sintered structure and longer plastic tensile deformation than sections at 30 min, which indicates that sufficient sintering between Ag particles has occurred to form joints with shear strengths up to 69.3 MPa. This is understandable because the increase in holding time helps the thermal decomposition of the organic matter in the Ag paste, which promotes the sintering diffusion between the Ag particles to form a high-density interconnected structure.

**Figure 15:**
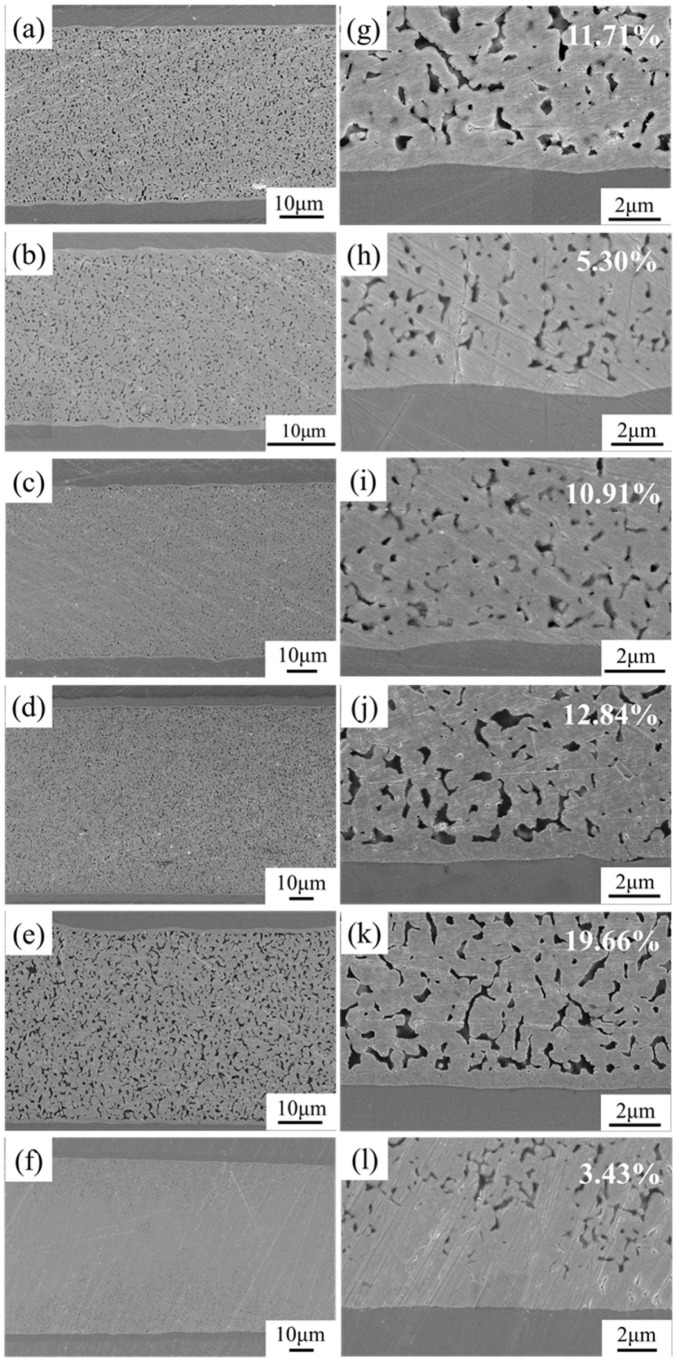
Cross-sections of pristine Ag pastes and different amine modified Ag paste bonded joints (**a**) Ag-0 (**b**) Ag-NOA (**c**) Ag-DDA (**d**) Ag-HDA (**e**) Ag-ODA (**f**) Ag-NOA holding for 60 min, (**g**–**l**) corresponding to partial enlargements of (**a**–**f**), respectively.

To investigate the relationship between the cross-sectional morphology of the joint and the bond strength, we prepared cross-sectional observation specimens by metallographic polishing method. Figure 15 shows the cross-sectional SEM characterization of the bonded specimens based on different Ag pastes. Figure 15g–i correspond to the partial high magnification features of Figure 15a–f, respectively. All joints held sintering for 30 min, except for Figure 15f,l, which present joints formed by holding under Ag-NOA sintering for 60 min. The pore size and porosity have a significant effect on the bonding performance of the joints, so we calculated the porosity of six types of joints using the Image-Pro Plus tool. In the unmodified case, there are obvious pores of different sizes in the cross-section of Ag-0 joints (Figure 15a,g) with a porosity of 11.71%, and the unevenly distributed pores lead to a reduction in shear strength. After the introduction of the lower boiling point NOA coating agent, it can be observed that large areas of Ag particles fuse together to form a uniform and continuous sintering network with a significant reduction in porosity to 5.3% and a very tightly bound Ag-Au interface (Figure 15b,h). The Ag-DDA joint also forms a denser sintered structure, but its pore size has slightly increased compared to Ag-NOA, and the porosity increases to 10.91%. Although a relatively dense sintered structure is formed in some regions of the Ag-HDA joint cross-section, large voids with 12.84% porosity are found by high magnification (Figure 15j), and the bonding line at the Ag-Au interface is also clearly visible, which indicates that the complete diffusion of nanoparticles at the binding interface is not formed. Interestingly, we find that the porosities of Ag-0, Ag-DDA, and Ag-HDA are similar, but the pore size of the latter two is significantly larger than that of the former, and these large pores can sprout and expand into cracks [52], leading to a decrease in shear strength. The Ag-ODA joint cross-section shows larger and more pores, and the sintered Ag structure is looser (Figure 15e). This may be due to the failure of the high boiling point ODA to volatilize and decompose sufficiently, and the organic residue prevents the sufficient diffusion of Ag particles. Meanwhile, the bonding connection at the Ag-Au interface is weaker near the interface (Figure 15k), and isolated Ag particles are present.

To investigate the effect of holding time on the cross-section of Ag-NOA joints, the holding time was increased to 60 min. Figure 15f,l show the cross-sectional morphology of the Ag-NOA joints at a holding time of 60 min. The morphology of the Ag particles can hardly be observed, and sufficient diffusion is achieved at the Ag–Au bonding interface. Figure 15f,l show the cross-sectional morphology of the Ag-NOA joints at a holding time of 60 min. The morphology of the Ag particles can hardly be observed, and sufficient diffusion is achieved at the Ag–Au bonding interface. Compared to the Ag-NOA joints at a holding time of 30 min, the porosity is slightly reduced to 3.43%. This can be explained here by the relationship between the diffusion coefficient and diffusion length as follows:

(1) $$D=D_{0}\exp\left(-\frac{Q_{d}}{RT}\right)$$

(2) $$L={\left(2D\times t\right)}^{\frac{1}{2}}\;\;\;\;\;\;\;$$

$\mathrm{where}\;D_{0}$, $Q_{d}$, R, T, and t are the temperature-independent preexponential, diffusion activation energy, gas constant, absolute temperature, and diffusion time, respectively. According to the equations, when the sintering holding time is extended from 30 min to 60 min, the diffusion length will only increase by 1.4 times, which is not a significant effect for sintering [53,54]. This indicates that increasing the holding time can enhance the sintering density of Ag-NOA joints to a lesser degree, which is verified by the small increase in shear strength.

**Figure 16:**
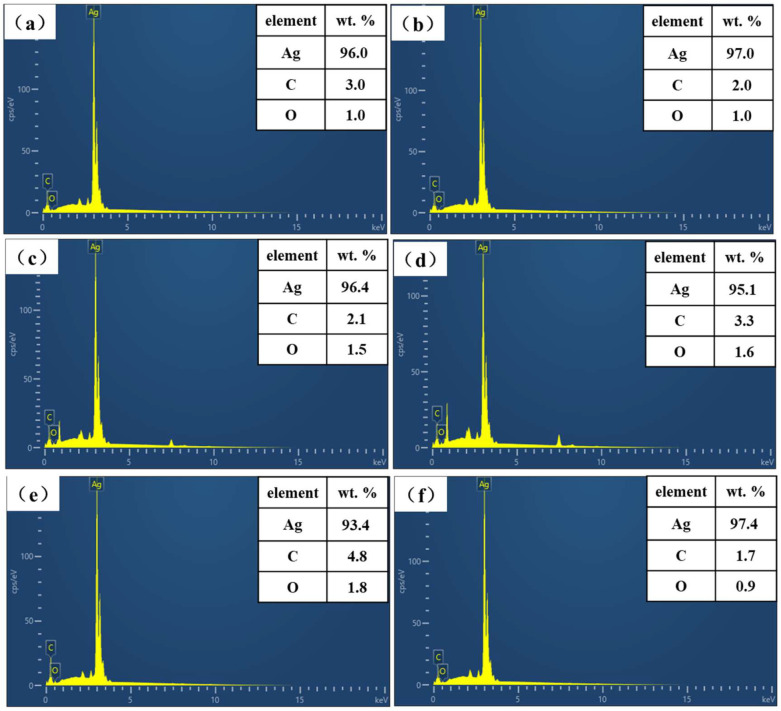
Cross-sectional EDS of unmodified and different amine modified Ag paste bonded joints (**a**) Ag-0, (**b**) Ag-NOA, (**c**) Ag-DDA, (**d**) Ag-HDA, (**e**) Ag-ODA and (**f**) Ag-NOA holding for 60 min.

The chemical compositions of different Ag bonded joint cross-sections were characterized by EDS energy spectra, as shown in Figure 16. The content of Ag in the joint is compared to determine the content of organic residue in the joint. The Ag contents in the cross-section of Ag-NOA and Ag-DDA joints are relatively higher with the Ag-0 joints, reaching 97.0 wt.% and 96.4 wt.% with relatively fewer organic residues at 30 min of holding time. However, the Ag content in the Ag-HDA and Ag-ODA joints is reduced relative to the Ag-0 joints, with values of 95.1 wt.% and 93.4 wt.%, respectively. It may be that with the increase in the number of carbon chains, the higher boiling point of organic amines is not easily decomposed and volatilized, which is not beneficial to the sintering and diffusion of Ag particles. When the holding time is increased to 60 min, the Ag content in the Ag-NOA joints slightly increases to 97.4 wt.%, which is similar to that of the Ag-NOA joints at a holding time of 30 min. This indicates that most of the organic matter in the Ag-NOA joint has been volatilized and decomposed at a holding time of 30 min.

### 3.5. Sintering Mechanism

**Figure 17:**
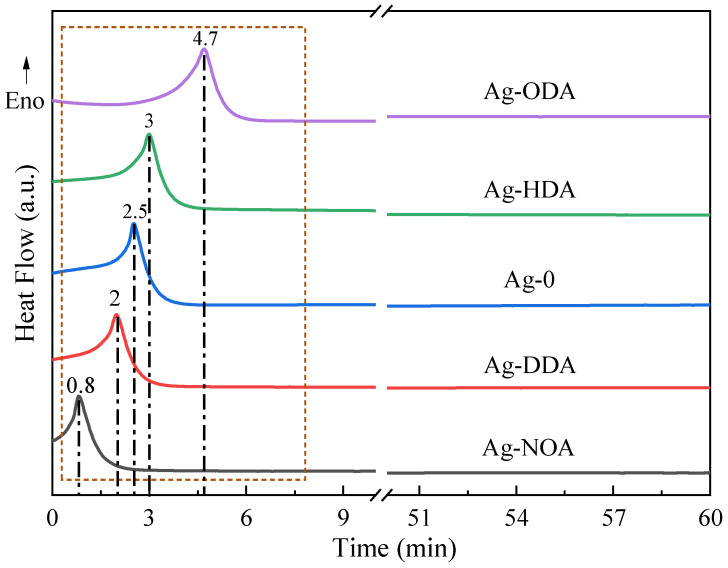
DSC curves of different amine modified Ag nanoparticles with holding time of 60 min at 250 °C.

Figure 17 shows the DSC curves of different amine modified Ag nanoparticles sintered at 250 °C with a holding time of 60 min, which helps to understand the sintering behavior of the Ag paste. Within the holding time of 5 min, exothermic peaks are observed for both unmodified Ag powders and different amine modified Ag powders. Interestingly, we find that the exothermic peaks of the four amine modified Ag nanoparticles appear successively with increasing boiling points. The NOA and DDA modified Ag particles show exothermic peaks at holding times of 0.8 min and 2 min, which are earlier than the appearance of exothermic peaks for unmodified Ag particles. However, the exothermic peaks of HDA and ODA modified Ag particles appear later than those of unmodified Ag particles. Based on the above analysis of the sintered Ag film and bond strength, we speculate that the earlier appearance of the exothermic peak facilitates deeper sintering diffusion between the Ag particles and improves the size and density of the sintered neck growth between the Ag particles. This is consistent with the results above that low boiling point amine modified Ag particle pastes have lower resistivity and higher joint strength.

**Figure 18:**
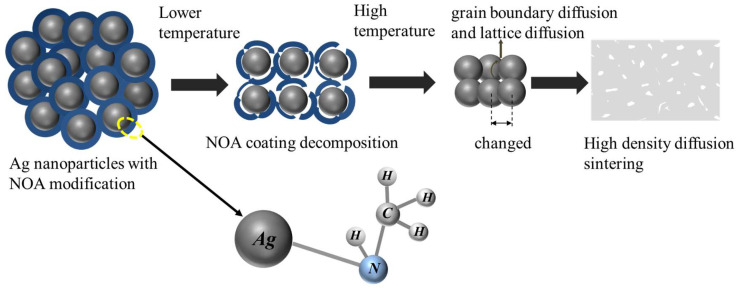
Sintering mechanism of NOA modified Ag nanoparticle paste.

The analysis based on the above results shows that the selection of suitable amine coating agents to modify the Ag nanoparticles helps to improve the sintering performance of the paste. In this paper, the sintering performance of amine modified Ag pastes with different boiling points is compared and NOA was found to have the best modification effect. Therefore, we speculate on the sintering mechanism, as shown in Figure 18. The whole sintering process can be divided into a low-temperature stage and a high-temperature stage. For the unmodified Ag particles, the exposed Ag nanoparticles with high surface activity will drive surface diffusion at room temperature or lower heating temperatures, resulting in a non-densified diffusion behavior and the formation of low-activity micron-sized agglomerated Ag structures. The non-densified diffusion behavior of nano-Ag in the low-temperature stage reduces the driving force required for densified diffusion. Therefore, it is difficult to obtain excellent sintering density when sintering in the high-temperature stage.

After modification by NOA, the –NH_2_ group undergoes a coordination reaction with Ag atoms to form Ag–N bonds attached to the surface of the particles. Due to the presence of the NOA coating layer, the Ag nanoparticles exhibit better dispersion and stability in the preparation of Ag paste and storage at room temperature. In the low-temperature stage, Ag nanoparticles do not directly trigger the sintering behavior, but first wait for the decomposition of low boiling point NOA on the particle surface. This limits the generation of low-density sintered structure formation of Ag nanoparticles at low heating temperature and promotes the main diffusion of Ag nanoparticles to change from surface diffusion to grain boundary diffusion and lattice diffusion. Smaller Ag nanoparticles with large surface-to-volume ratio can provide enough energy to stimulate grain boundaries and lattice diffusion, which can form a large, sintered grain size and high-density sintered structures at high temperature stage.

In addition, the other three higher boiling point amine modified Ag particles take longer to decompose the coating agent when sintered at 250 °C, and even too much organic matter can remain. In particular, residual organic matter can weaken the sintering diffusion between Ag particles and inhibit the densification of Ag sintering.

Based on the above analysis, it can be concluded that the commercial Ag nanoparticles can be effectively modified and the pressureless sintering performance of the Ag pastes can be enhanced by selecting the appropriate organic amine with the proper treatment process. The Ag paste prepared by using *n*-octylamine modified Ag nanoparticles has low resistivity and high bonding strength. Its surface morphology is uniform and dense, consisting of larger silver grains and fewer organic residues.

## 4. Conclusions

In this paper, a novel surface treatment process based on commercial Ag nanoparticles is proposed that can prevent the agglomeration of Ag nanoparticles and enhance the Ag paste sintering performance. This method is simple, efficient, and time-saving to operate. Four types of amine modified Ag particles with different boiling points are used to prepare pastes, the effect of amine type on the sintering performance is investigated, and its application in pressureless die attachment is discussed. The results show that the -NH_2_ group in the organic amine adsorbs on the surface of Ag particles, forming an organic coating layer and preventing the agglomeration of Ag particles at room temperature. The sintering properties of Ag pastes are closely related to the amine used, since the amine influences the thermal decomposition of the Ag particles, resulting in different grain sizes, sintering structures, and organic residues. The grain size and sintering density after sintering impact the resistivity of the film, with larger grains and higher density improving the electrical conductivity of the film. Ag-NOA has the lowest resistivity of 7.31 μΩ·cm at 250 °C with 30 min holding time, and the result is significantly better than that of the unmodified film. After increasing the holding time to 60 min, the resistivity slightly decreased to 5.3 μΩ·cm. The micro-morphology, porosity, and organic residues of sintered Ag affect the shear strength of bonded samples. The Ag-NOA joint has a bond strength of up to 61.8 MPa at 250 °C with 30 min holding time. The fracture surface shows a clearly plastic deformation structure. The cross section shows a dense sintered network with the lowest porosity and the highest interface connectivity. The EDS energy spectra showed the least organic residues after Ag-NOA sinter bonding. Increasing the holding time can promote the full diffusion sintering of Ag particles. The shear strength was slightly increased to 69.3 MPa at 250 °C with 60 min holding time. In addition, we have proposed the corresponding sintering mechanism based on the excellent sintering effect achieved by NOA-modified Ag nanoparticle paste, explaining the influence of the organic amine modification on the sinter formation of Ag nanoparticles in the low-temperature stage and high-temperature stage. The study provides effective experimental data and theoretical support for pressureless sintering technology, which has good potential for practical application in power device packaging.

## Figures and Tables

**Table 1:** Particle size of nano-Ag crystals in sintered films of different organic amine modified Ag pastes.

Ag Paste	2θ (°)	FWHM	Size (nm)
Ag-0	38.07556	0.24636	35.6559
Ag-NOA	38.2275	0.15977	55.00541
Ag-DDA	38.00677	0.19828	44.29276
Ag-HDA	38.1224	0.26087	33.67741
Ag-ODA	38.04315	0.36849	23.836

**Table 2:** Particle size of nano-Ag crystals in Ag-NOA sintered films at different holding times.

Time (min)	2θ (°)	FWHM	Size (nm)
10	38.1475	0.25118	34.97926
30	38.2275	0.15977	55.00541
60	38.2751	0.14933	58.85945

## Data Availability

The data that support the findings of this study are available from the corresponding author upon reasonable request.

## Supplementary Materials

The following supporting information can be downloaded at: https://www.mdpi.com/article/10.3390/nano12193351/s1, Figure S1: Heating curve of sintering process; Figure S2: XRD diffractograms of the organic amine modified Ag nanoparticles; Figure S3: Shear strength of different organic amine modified nano-Ag pastes sintered at 250 °C for different holding times.
