# Supplementary material for: High Strength Die-Attach Joint Formation by Pressureless Sintering of Organic Amine Modified Ag Nanoparticle Paste
Source: Nanomaterials (Basel). 2022 Sep 26;12(19):3351. doi: 10.3390/nano12193351 (PMC9565494; doi:10.3390/nano12193351)
Supplement: Supplementary file 1 [file nanomaterials-12-03351-s001.zip › nanomaterials-1902824-supplementary.pdf]

Supplementary Materials

High Strength Die-Attach Joint Formation by Pressureless Sintering of Organic Amine Modified Ag Nanoparticle Paste

Xingwang Shen [1,2,†], Junjie Li [2,†] and Shuang Xi [1,*]

[1] College of Mechanical and Electronic Engineering, Nanjing Forestry University, Nanjing 210037, China
[2] Shenzhen Institute of Advanced Electronic Materials, Shenzhen Institute of Advanced Technology, Chinese Academy of Science, Shenzhen 518100, China
* Correspondence: shuangxi@njfu.edu.cn
† These authors contributed equally to this work.

Figure S1 shows the heating curve of the pressureless sintering process of the samples in this paper. The samples were heated from 30°C to 250°C at a steady rate of 16°C/min in the atmosphere, held for different durations (10min, 30min or 60min), and then cooled down naturally.

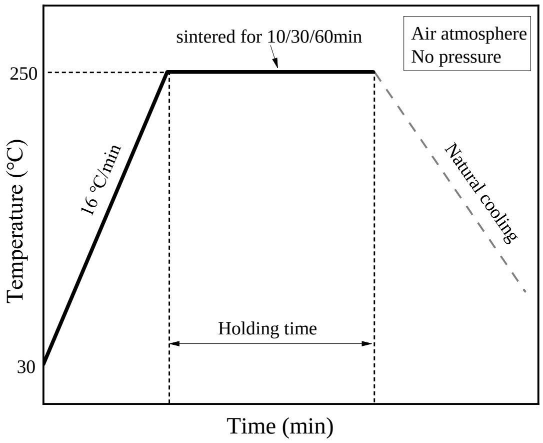

Figure S1. Heating curve of sintering process

Figure S2 shows the XRD diffractograms of the organic amine modified Ag nanoparticles. Five major diffraction peaks appear in the figure, namely (111), (200), (220), (311) and (222), which correspond to the crystalline plane of the pure Ag phase. These XRD diffraction peaks are consistent with the standard powder diffraction card (JCPDS No. 03-065-2871) for Ag face-centered cubic crystal structure. Meanwhile, we did not find the diffraction peaks of AgO, Ag2O and other impurities.

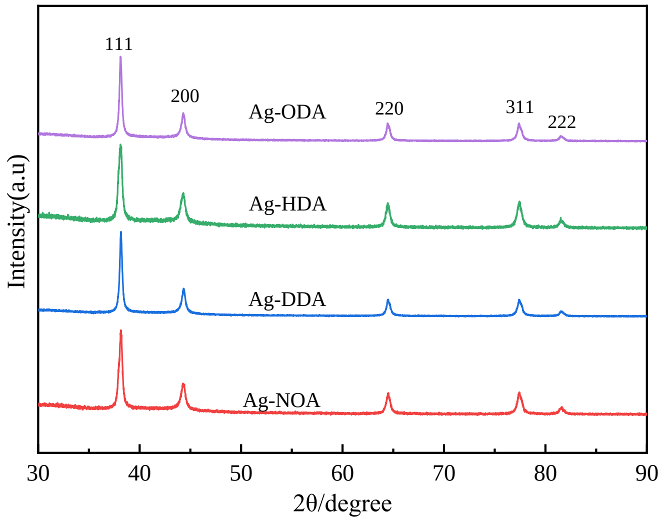

Figure S2. XRD diffractograms of the organic amine modified Ag nanoparticles

Figure S3 shows the shear strength of different organic amine modified nano-Ag pastes sintered at 250 °C for different time. The bonding strength of each type of Ag paste joint increases with increasing holding time. The shear strength of Ag-0 joints does not change significantly with time, which may be due to the decrease in surface energy caused by nano-Ag agglomeration. Ag-NOA joints can reach 45.7 MPa after 10 min of holding time. When the holding time is increased to 30 min, the joint strength can reach 61.8 MPa. The joint strength reached the highest value of 69.3 MPa in this paper at the holding time of 60 min. This indicates that extending the holding time of Ag-NOA can realize improved sintering state. The joint strengths of Ag-DDA, Ag-HDA and Ag-ODA are all lower than those of Ag-0 at the holding time of 10 min, which may be resulted from that the coating agent has not been completely decomposed and the organic residues hinder the sintering of the Ag particles. At the holding time of 30 min, the shear strength of all three joints of Ag-DDA, Ag-HDA and Ag-ODA are increasing. In particular, the strength of Ag-DDA joints is much higher than that of Ag-0. When the holding time is increased to 60 min, the shear strength of Ag-DDA joint grows smaller, while the shear strength of Ag-HDA and Ag-ODA joints still shows intense growth. This may be due to that the coating agent in Ag-DDA has decomposed relatively sufficiently at 30 min, and continuing to increase the holding time can promote a more adequate sintering of the Ag particles. While there still exist considerable organic residues in the Ag-HDA and Ag-ODA joints even after 60 min holding duration, thus resulting in lower shear strength.

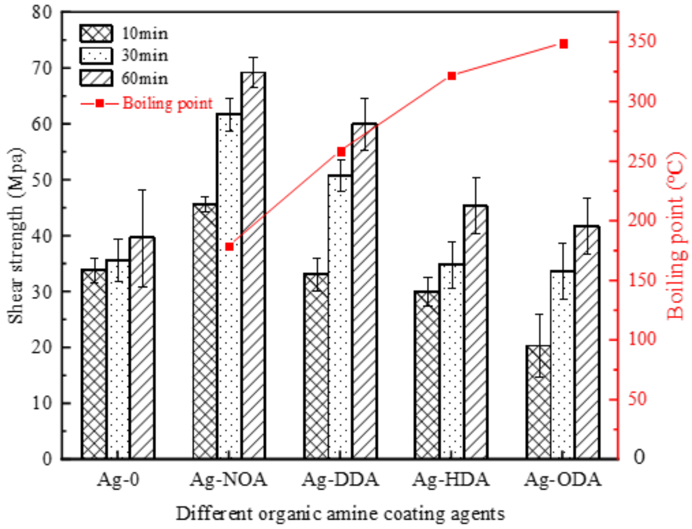

Figure S3. Shear strength of different organic amine modified nano-Ag pastes sintered at 250 °C for different holding times
